# Human Lymphocyte-Protective Effects of an Ethanol Extract from *Detarium microcarpum* Guill. and Perr. (Caesalpiniaceae) Fruit Pulp

**DOI:** 10.3390/antiox7080104

**Published:** 2018-08-04

**Authors:** Ablassé Rouamba, Moussa Compaoré, Maurice Ouédraogo, Martin Kiendrebeogo

**Affiliations:** 1 Laboratory of Applied Biochemistry and Chemistry (LABIOCA), UFR-SVT, University Ouaga 1 Pr Joseph KI-ZERBO, 03 BP 7021 Ouagadougou 03, Burkina Faso; rouambaablasse@gmail.com (A.R.); martinkiendrebeogo@yahoo.co.uk (M.K.); 2 Laboratory of Animal Physiology (LPA), UFR-SVT, University Ouaga 1 Pr Joseph+ KI-ZERBO, 09 BP 848, Ouagadougou 09, Burkina Faso; ouedraogo_maurice@yahoo.fr

**Keywords:** antioxidant, cytoprotective, hydrogen peroxide, tert-butyl hydroperoxide

## Abstract

The current study aimed to evaluate, in vitro, the antioxidant capacity and the human lymphocyte-protective effect of the ethanolic extract from *Detarium microcarpum* fruit pulp against oxidative stress damage. Human lymphocytes were incubated with different concentrations of extract, followed by the addition of hydrogen peroxide or tert-butyl hydroperoxide. Cell viability was measured using 3-(4,5-dimethylthiazol-2-yl)-2,5-diphenyltetrazolium bromide (MTT) assay. Furthermore, the antioxidant property of the extract was evaluated in vitro using hydrogen peroxide and nitric oxide radical-scavenging assays. Compared to the vehicle, the fruit pulp ethanol extract did not exhibit a cytotoxic effect on human lymphocytes. Furthermore, the cytotoxicity of hydrogen peroxide and tert-butyl hydroperoxide to human lymphocytes was significantly reduced by fruit extract pretreatment. The extract and ascorbic acid exhibited similar cytoprotective activity (*p* > 0.05). The fruit pulp extract showed more antioxidant activity than gallic acid in the hydrogen peroxide-scavenging model, while in the nitric oxide-quenching model, the fruit extract and gallic acid showed similar activity. The fruit pulp of *D. microcarpum* contains potent antioxidant and cell-protective compounds. The use of the fruit pulp of *D. microcarpum* as a food supplement could rescue cellular oxidative damage responsible for numerous pathologies.

## 1. Introduction

Several studies have established a relationship between oxidative stress and the human immunity deficiency, because it alters biological molecules of lymphocytes such as DNA, biomembranes, and proteins. This leads to the cellular injury and death that causes the development of numerous diseases and disorders such as aging, cancer, atherosclerosis, diabetes, cirrhosis, and cataracts [1,2]. Tert-butyl hydroperoxide (t-BOOH) and hydrogen peroxide have been reported to induce oxidative stress in different organs including the liver, testes, oocytes, retina, and lymphocytes [3,4]. Under the cytotoxic condition, it acts mainly by mobilization of arachidonic acid from membrane phospholipids. The consequences are the augmentation of the intracellular arachidonic acid level, malondialdehyde formation, and alteration in cellular calcium and glutathione levels, resulting in cellular oxidative damage [5,6]. Bioactive compounds derived from edible plants capable of quenching free radicals or preserving cell membrane integrity are of great interest in combating oxidative stress-induced cellular damage [7,8]. So, plant bioactive compounds such as phenolics, flavonoids, anthocyanins, and vitamins may exert various biological activities on cells [8,9]. These compounds could prevent free-radical-mediated cellular oxidative damage by quenching the free radicals and by trapping the free metals. In addition, the polyphenols of fruits could modulate the membrane permeability and limit the incorporation of deleterious compounds that could affect cell membrane integrity [8].

*Detarium microcarpum* (Caesalpiniaceae) is widely and commonly used in traditional medicine to treat diverse ailments including malaria, bronchitis, and meningitis [9,10,11]. Bioactive diterpenes with antifungal, antioxidant, and neuroprotective activities were isolated from its fruit pulp extract [12]. Phenolic compounds such as flavonoids and anthocyanins were also characterized in this fruit pulp extract [13]. Finally, the high nutritional values of the fruit pulp are well documented, and it is commonly used in west African regions as a human diet supplement [14,15].

The present investigation aims to evaluate the cell-protective property of the ethanolic extract from *Detarium microcarpum* fruit pulp against hydrogen peroxide- and tert-butyl hydroperoxide-induced human lymphocyte cytotoxicity in vitro. Furthermore, the antioxidant capacity of the extract to scavenge hydrogen peroxide and nitric oxide radicals was evaluated. 

## 2. Materials and Methods

### 2.1. Plant Material Collection and Extraction

The fresh fruits of *D. microcarpum* were collected in Gampela at a site situated 25 km east of Ouagadougou (Burkina Faso) in January 2015. The sample’s identity was certified by Jeanne Millogo-Rasolodimby, a botanist from the Laboratory of Plant Biology and Ecology (Université Ouaga I Pr Joseph KI-ZERBO, Ouagadougou, Burkina Faso). The identification code of the herbaria specimen is 15928. The fresh fruits were washed with distilled water, and fruit pulp was dried at room temperature (25 °C) and powdered. To minimize the degradation of thermolabile compounds, extraction of 25 g of pulp powder was conducted by maceration (25 °C) in ethanol over 24 h under continuous stirring. The extract was filtrated and concentrated to dryness in a vacuum evaporator and stored at 4 °C until further investigation.

### 2.2. Chemicals and Reagents

Gallic acid, sodium nitroprusside, Griess reagent for nitrite, 3-(4,5-dimethylthiazol-2-yl)-2,5-diphenyltetrazolium bromide (MTT), dimethyl sulfoxide, RPMI 1640, tert-butyl hydroperoxide, Histopaque-1077, trypan blue, phosphate buffer saline, gentamycin, and fetal bovine serum were purchased from Sigma-Aldrich (St. Louis, MO, USA). Hydrogen peroxide, ascorbic acid, and ethanol were supplied by Labosi (Paris, France). 

### 2.3. Antioxidative Activity

#### 2.3.1. Nitric Oxide Scavenging Assay

The capacity of the extract to scavenge nitric oxide radicals was performed as described previously [16]. For the experimental design, 100 µL of sodium nitroprusside (10 mM) dissolved in phosphate-buffered saline (50 mM; pH: 7.4) was mixed with the same amount of different concentrations of extract (6.5–300 µg/mL) dissolved in phosphate-buffered saline (PBS). The mixture was incubated at room temperature for 2 h. The vehicle was constituted by the same reaction mixture, without extract, but with an equivalent amount of PBS. After the incubation period, 100 µL of Griess reagent was added to the mixture, and the absorbance of the formed chromophore resulting in the reaction of free nitric oxide radicals and Griess reagent was measured spectrophotometrically at 546 nm using a microplate reader (BioTek Instruments, ‎Winooski, VT, USA). The percentage of nitric oxide radicals scavenged by each concentration of extract was calculated, and the concentration of extract (µg/mL) scavenging 50% of nitric oxide radicals (IC_50_) was determined. Gallic acid and ascorbic acid were used as standards, and all experiments were conducted in triplicate. 

#### 2.3.2. Hydrogen Peroxide Scavenging Assay

The capacity of the extract to scavenge hydrogen peroxide was examined as described previously [17]. The solution of hydrogen peroxide (100 mM) was prepared in phosphate buffer (50 mM; pH: 7.4). One hundred microliters of extract at different concentrations (6.5–300 µg/mL) in PBS was added to the same amount of hydrogen peroxide solution (100 mM). The vehicle was maintained by mixing 100 µg of the hydrogen peroxide solution with the same amount of PBS without extract. After 10 min of incubation at room temperature, the absorbance of unreactive hydrogen peroxide was determined at 230 nm against a blank solution containing phosphate buffer without hydrogen peroxide. The percentage of hydrogen peroxide scavenged by each concentration of extract tested was calculated, and the concentration of extract (µg/mL) scavenging 50% of the hydrogen peroxide (IC_50_) was determined. Gallic acid and ascorbic acid were used as standards, and all experiments were conducted in triplicate. 

### 2.4. Cytoprotective Activity

#### 2.4.1. Blood Collection and Lymphocyte Isolation and Culture

Peripheral blood samples obtained from healthy volunteer donors with an average age of 28 that did not smoke, drink, or use chronic medication were collected after 12 h overnight fasting by venipuncture using a top Vacutainer^®^ (BD Diagnostics, Plymouth, UK). The study was approved by the Ethic Committee of the Regional Center of Blood Transfusion of Ouagadougou (Burkina Faso) under the reference agreement no. 2015-340/MS/SG/CNTS/CRTS-O. 

Peripheral blood lymphocytes were isolated under sterile conditions by using a density gradient present in the Histopaque-1077 reagent, according to the study by Gafrikova et al. [18]. Five milliliters of blood were mixed in Eppendorf tubs with the same volume of phosphate buffer solution. Histopaque-1077 (100 μL) was underlayed and tubs were spun at 800× *g* for 30 min at 4 °C. Lymphocytes were retrieved from just above the boundary between the phosphate buffer and Histopaque-1077. One hundred microliters of lymphocytes were mixed with 1 mL of PBS in new Eppendorf tubes and spun again at 800× *g* for 5 min at 4 °C. Supernatant was removed and the lymphocytes were counted in a Neubauer chamber using Trypan blue (0.4%). Cell viability of about 98% was accepted for further experiments.

Lymphocytes were cultured in culture medium containing 1 mL RPMI 1640 supplemented with 10% of fetal bovine serum and 1 mM of gentamycin. Cells were maintained in a suspension culture at 37 °C in a humidified 5% CO_2_ atmosphere.

#### 2.4.2. Cell Treatment and MTT Assay

To assess the effect of fruit pulp extract on human lymphocyte viability (cytotoxicity), cells were incubated in 96-well plates (5 × 10^3^ cells /well) for 1 h with vehicle (culture medium RPMI) or with different concentrations of extract (100–1000 µg/mL), and the percentages of viable cells in the extract treatment were compared to the vehicle. Furthermore, to evaluate the potential of the extract to protect human lymphocytes from oxidative damage (cytoprotection), cells were incubated (1 h, 37 °C, 5% CO_2_) with samples at different concentrations (100–1000 µg/mL) before exposure to the hydrogen peroxide or to tert-butyl hydroperoxide cytotoxic actions. The 3-(4,5-dimethylthiazol-2-yl)-2,5-diphenyltetrazolium bromide (MTT) assay was performed as described previously for evaluating the cytotoxicity and the cytoprotective effects of the extract [19]. Briefly, fresh medium (100 μL) containing MTT (0.5 mg/mL) was added into each well containing pretreated cells and incubated for 2 h at 37 °C. After removing MTT-containing medium, 100 μL of dimethyl sulfoxide (DMSO) was added into each well to dissolve the purple formazan crystal formed in viable cells. The plates were then shaken gently for 20 min in darkness and absorbance was measured at 595 nm on a microtiter plate reader. Ascorbic acid was used as the standard and the experiment was performed in triplicate. 

### 2.5. Statistical Analysis

The experiments were conducted in triplicate (*n* = 3) and data presented as mean values ± standards deviation. GraphPad software (GraphPad Software Inc, San Diego, CA, USA) was used to produce graphs and curves. Statistical analysis was assessed by using one-way ANOVA for repeated measures followed by Newman–Keuls post-test. *p* values < 0.05 were considered as being significant. 

## 3. Results

### 3.1. Antioxidant Activity

The antioxidant property of the fruit pulp ethanol extract was examined through its capacity to scavenge hydrogen peroxide and nitric oxide radicals in vitro. The results are presented in Table 1. It was found that the extract exhibited high scavenging activities for hydrogen peroxide and nitric oxide radicals, with IC_50_ values of 90.21 ± 2.07 µg/mL and 25.50 ± 0.01 µg/mL, respectively. Interestingly, the extract showed higher hydrogen peroxide scavenging activity (IC_50_ = 90.21 ± 2.07 µg/mL) than gallic acid (IC_50_ = 109.98 ± 2.15 µg/mL), while in the nitric oxide radical quenching model, the extract and gallic acid exhibited similar activity (*p* > 0.05). Ascorbic acid showed the highest antioxidant activity. These findings suggested that the ethanol extract of fruit pulp from *D. microcarpum* is an effective scavenger of free radicals. 

### 3.2. Cytoprotective Activity

**Figure 1:**
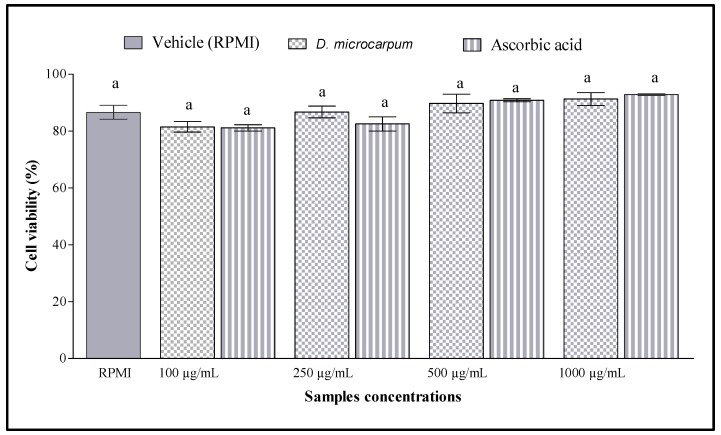
Cytotoxic effect of ethanol extract and ascorbic acid. ^a^ Values with the same superscripted letters do not differ significantly (*p* > 0.05) as determined by analysis of variance.

**Figure 2:**
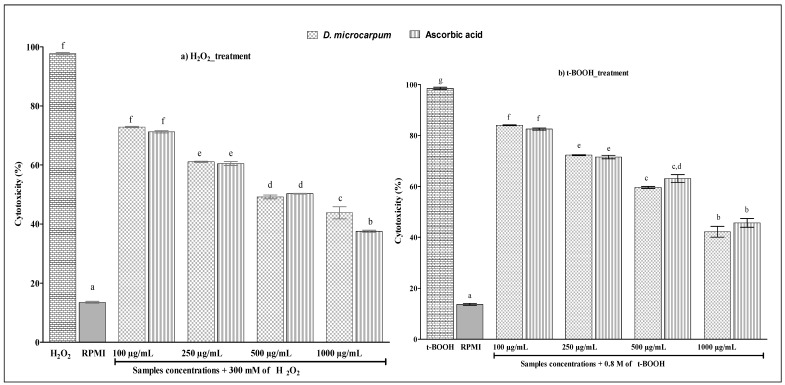
Cytoprotective effect of ethanol extract and ascorbic acid: (**a**) against hydrogen peroxide-induced human lymphocyte oxidative damage; (**b**) against tert-butyl hydroperoxide-induced human lymphocyte oxidative damage. Values are expressed as mean values ± standards deviation (*n* = 3 independent experiments). ^a–g^ Values with different superscripted letters for each oxidant applied differ significantly (*p* < 0.05) as determined by analysis of variance.

The human lymphocyte viability data are shown in Figure 1. It was found that the extract did not significantly affect the integrity of human lymphocytes. There was no cytotoxic effect of the extract in the range of concentrations tested. The cytoprotection of samples is shown by Figure 2. A substantial cytotoxicity was observed when cells were treated with hydrogen peroxide alone or with tert-butyl hydroperoxide alone. However, the cytotoxicity of hydrogen peroxide and tert-butyl hydroperoxide decreased gradually with the increasing of extract’s concentrations. The same results were observed with the standard, ascorbic acid. Interestingly, the extract and ascorbic acid exhibited similar cytoprotective action (*p* > 0.05). Moreover, the extract at 1000 µg/mL reduced more than 50% of the cytotoxicity of hydrogen peroxide and tert-butyl hydroperoxide*.*

## 4. Discussion

The importance of fruit consumption in relation to human health has increased the interest in research on bioactive components in food. This present investigation demonstrates the importance of the use of wild fruits as supplements in the human diet for health care. The use of fruits as food supplements could be a good strategy to prevent the chronic diseases associated with cellular oxidative damage. These findings demonstrate that the ethanol extract of the fruit pulp of *D. microcarpum* is a potent free radical fighter as well as a potent cell protector. The potential of the extract to quench hydrogen peroxide and nitric oxide radicals in vitro could justify its cytoprotective action against hydrogen peroxide and tert-butyl hydroperoxide. Several mechanisms could be implicated in the processes of the cytoprotective effect of the extract, such as the direct scavenging of free radicals (hydroxyl radicals, butoxyl radicals, and nitric oxide) generated by the oxidative stress induced by hydrogen peroxide and tert-butyl hydroperoxide, the increase of intracellular levels of antioxidant enzymes and glutathione, or the reduction of hydrogen to water molecules. The free radical scavenging potential of fruit pulp of *D. microcarpum* demonstrated in this study was in accord with previous studies [20]. The human lymphocyte-protective activity of ascorbic acid against hydrogen peroxide was previously demonstrated [21]. The fruit pulp of *D. microcarpum* was reported to possess high ascorbic acid content, which could contribute to its cytoprotective activity as observed in this present investigation [15]. Moreover, in our previous studies, the ethanol extract of fruit pulp of *D. microcarpum* showed, in vitro, antihemolytic activities in human and rat erythrocytes [22]. All these findings demonstrate that the fruit pulp of *D. microcarpum* is a potent cell protector against oxidative damage and justify the traditional use of this fruit pulp in the treatment of Alzheimer’s disease caused by the oxidative damage of neurons [12]. Previous studies have demonstrated the cell-protective activities of bioactive compounds from edible fruits. Polyphenols found in blueberries showed a cytoprotective activity against t-BOOH-induced cellular oxidative damage in rat hepatocytes [23]. Bioactive flavonoids of grapefruit (*Citrus paradisi* Macf.) such as naringin and isonaringin exerted a strong cytoprotective effect on SH-SY5Y neuroblastoma cell lines at concentrations ranging within 100–250 µg/mL [24]. Flavones and C-glucosydes of *Jatropha curcas* such as vitexin, vicenin-2, stellarin-2, and rhoifolin showed cytoprotective activity on human lymphocytes exposed to t-BOOH [25]. Phytochemical screening of the fruit pulp of *D. microcarpum* may promote the establishment of future food additives for the benefit of consumer health. 

## 5. Conclusions

This study highlighted the antioxidant and cytoprotective properties of the fruit pulp extract of *D. microcarpum.* According to the interesting antioxidant and cytoprotective activities of fruit pulp of *D. microcarpum* observed in this study, the use in human nutrition could prevent cellular oxidative damage responsible for numerous pathologies. Further investigations will involve the isolation and identification of cytoprotective compounds from this fruit pulp. 

## Figures and Tables

**Table 1:** Antioxidant activity of fruit pulp ethanol extract from *Detarium microcarpum* and standard compounds.

Concentrations (µg/mL)	H_2_O_2_ Scavenging Activity (%)			Radical NO Scavenging Activity (%)		
	Ethanol Extract	Gallic Acid	Ascorbic Acid	Ethanol Extract	Gallic Acid	Ascorbic Acid
6.5	29.38 ± 2.12 ^a^	10.49 ± 0.52 ^c^	19.4 ± 0.24 ^b^	19.93 ± 1.39 ^b^	41.28 ± 2.47 ^a^	43.02 ± 0.82 ^a^
12.5	40.22 ± 0.77 ^a^	12.56 ± 0.77	23.02 ± 0.29 ^b^	40.96 ± 0.85 ^c^	46.69 ± 0.43 ^b^	54.52 ± 1.28 ^a^
25	45.86 ± 0.49 ^a^	17.59 ± 1.01 ^c^	30.40 ± 0.44 ^b^	49.40 ± 0.85 ^c^	52.11 ± 1.28 ^b^	55.72 ± 0.43 ^a^
50	45.76 ± 3.01 ^b^	20.26 ± 1.12 ^c^	68.49 ± 4.25 ^a^	54.57 ± 2.16 ^b^	54.88 ± 1.72 ^b^	57.62 ± 0.43 ^a^
75	46.66 ± 0.84 ^b^	28.08 ± 3.2 ^c^	69.58 ± 0.73 ^a^	66.57 ± 3.83 ^c^	70.18 ± 0.43 ^a^	68.67 ± 0.85 ^b^
150	61.56 ± 3.56 ^c^	68.59 ± 1.04 ^b^	79.06 ± 0.34 ^a^	65.66 ± 0.85 ^c^	78.61 ± 0.43 ^a^	71.99 ± 0.43 ^b^
300	61.72 ± 0.39 ^c^	85.44 ± 2.59 ^a^	79.26 ± 0.19 ^b^	72.38 ± 0.41 ^c^	84.64 ± 0.43 ^a^	78.61 ± 1.28 ^b^
IC_50_ (µg/mL)	90.21 ± 2.07 ^b^	109.98 ± 2.15 ^c^	39.55 ± 2.35 ^a^	25.50 ± 0.01 ^b^	23.57 ± 1.71 ^b^	10.28 ± 1.05 ^a^

Values are expressed as mean values ± standards deviation (*n* = 3 independent experiments). ^abc^ Values within each line with different superscripted letters for each antioxidant assay differ significantly (*p* < 0.05) as determined by using analysis of variance. IC_50_: inhibitor concentration giving 50% reduction of radicals.
